# The synthetic phospholipid C8-C1P determines pro-angiogenic and pro-reparative features in human macrophages restraining the proinflammatory M1-like phenotype

**DOI:** 10.3389/fimmu.2023.1162671

**Published:** 2023-06-16

**Authors:** Juan Manuel Ortiz Wilczyñski, Hebe Agustina Mena, Martin Manuel Ledesma, Cinthia Mariel Olexen, Enrique Podaza, Mirta Schattner, Soledad Negrotto, Andrea Emilse Errasti, Eugenio Antonio Carrera Silva

**Affiliations:** ^1^ Institute of Experimental Medicine, National Scientific and Technological Research Council - National Academy of Medicine (IMEX-CONICET-ANM), Buenos Aires, Argentina; ^2^ Institute of Pharmacology, School of Medicine, University of Buenos Aires, Buenos Aires, Argentina; ^3^ Caryl and Israel Englander Institute for Precision Medicine, Weill Cornell Medicine, New York, NY, United States

**Keywords:** ceramide 1-phosphate, macrophages, inflammation, tissue repair, angiogenesis

## Abstract

Monocytes (Mo) are highly plastic myeloid cells that differentiate into macrophages after extravasation, playing a pivotal role in the resolution of inflammation and regeneration of injured tissues. Wound-infiltrated monocytes/macrophages are more pro-inflammatory at early time points, while showing anti-inflammatory/pro-reparative phenotypes at later phases, with highly dynamic switching depending on the wound environment. Chronic wounds are often arrested in the inflammatory phase with hampered inflammatory/repair phenotype transition. Promoting the tissue repair program switching represents a promising strategy to revert chronic inflammatory wounds, one of the major public health loads. We found that the synthetic lipid C8-C1P primes human CD14^+^ monocytes, restraining the inflammatory activation markers (HLA-DR, CD44, and CD80) and IL-6 when challenged with LPS, and preventing apoptosis by inducing BCL-2. We also observed increased pseudo-tubule formation of human endothelial-colony-forming cells (ECFCs) when stimulated with the C1P-macrophages secretome. Moreover, C8-C1P-primed monocytes skew differentiation toward pro-resolutive-like macrophages, even in the presence of inflammatory PAMPs and DAMPs by increasing anti-inflammatory and pro-angiogenic gene expression patterns. All these results indicate that C8-C1P could restrain M1 skewing and promote the program of tissue repair and pro-angiogenic macrophage.

## Introduction

The immune system is a critical component of the many processes involved in tissue injury and repair. Upon injury, local epithelial and endothelial cells and fibroblasts, as well as resident tissue leukocytes (such as macrophages) undergo significant activation and proliferation, releasing chemokines and inflammatory cytokines. These soluble factors immediately recruit circulating monocytes and neutrophils to the affected site initiating the tissue repair cascade thus promoting tissue restoration (1, 2). The magnitude and duration of the inflammatory response depend on an adequate integration of resolutive signals to reach homeostasis again. In chronic wounds and fibrosis, aberrant repair signaling predominates with unresolved inflammation. Resident macrophage populations are critical in maintaining homeostasis under steady-state conditions, while monocyte-derived macrophage populations are recruited from the bone marrow in large numbers after injury and are critical to restoring equilibrium. Macrophages can differentiate into a plethora of subtypes depending on the local tissue environment, and their phenotype and functions are shaped by the local metabolites, growth factors, and cytokines. Moreover, macrophages are also the main producers of resolutive factors to dampen inflammation and induce re-vascularization and tissue repair. Thus, the different stages of tissue repair must be carefully regulated given that macrophages of different phenotypes play unique and critical roles at each stage (3).

Natural phospho-sphingolipids represent one kind of the many lipid metabolites that are released under inflammatory conditions. While sphingosine-1-phosphate (S1P) is constitutively produced from healthy cells, the related sphingolipid ceramide-1-phosphate (C1P) is instead highly produced by stressed and dead cells. At some point, C1P was considered a damage-associated molecular pattern (DAMP) that rapidly increases its levels in injured tissues. These and other sphingolipid-derivatives have been considered for many years as simple structural cell components, but they undoubtedly are powerful bioactive lipids that impact several cellular targets to trigger an innate response that mostly consists of attracting immune and non-immune effector cells and inducing the proliferation of fibroblasts, myoblasts, and stem cells (4–6).

While both phospho-sphingolipids C16-C1P and C8-C1P (a natural long-chain ceramide and a shorter synthetic analog, respectively) are well known to have chemoattractant, antiapoptotic, and mitogenic activity (7, 8), in a previous study we have reported that local administration of C8-C1P alone enhanced leg reperfusion and muscle regeneration in a mouse model of hindlimb ischemia (9). Furthermore, when C8-C1P intramuscular injection was combined with human endothelial colony forming cells (hECFCs) infusion or hECFCs previously preconditioned with C8-C1P, the reperfusion index, and the preserved functional tissue increased, together with a reduction on the necrotic area of the ischemic muscle (9). In this sense, C8-C1P has a great potential to be therapeutically used for many diseases that compromise immune system dysregulation, ischemia, or tissue damage (e.g., ulcerative colitis, sepsis, cigarette-induced chronic obstructive pulmonary disease, asthma, and ischemic affections, among others) (10–12). Circulating monocytes are among the first wave of leukocytes to infiltrate injured tissue, initially contributing to the local inflammation but later the monocyte-derived macrophages with resolutive and tissue repair properties are required for to clear of dead cells, dampen inflammation, and induce re-vascularization by stimulating migration, proliferation, and differentiation of hECFCs (3, 9, 13). In this context, we focused our work on the priming or programming of human CD14 monocytes when challenged with C8-C1P sphingolipid, conditioning not only the acute response but also their monocyte-derived macrophage (MDM) differentiation and their effector response. In addition, we have also tried to mimic some local tissue inflammatory conditions as the exposure to lipopolysaccharide (LPS) from Gram-negative bacteria, apoptotic/necrotic neutrophils, lipoteichoic acid (LTA) from Gram-positive bacteria, as well as low molecular weight hyaluronic acid (HA), all of them commonly found in exposed injuries, to compare the strength of C8-C1P signaling to keep a resolutive program. Our results could represent a promising strategy to revert chronic inflammatory wounds.

## Materials and methods

### Lipid preparation, PAMPs, and DAMPs utilization

C8-C1P d18:1/8:0 (C8-C1P) and C16-C1P d18:1/16:0 (C16-C1P) were purchased from Avanti Polar Lipids, Inc, Alabaster, AL. These lipid products were prepared according to the manufacturer’s instructions in ultrapure water by sonication on ice, using a probe sonicator until a clear dispersion was observed. The concentration used in this work was between 1 to 20 µM. Sonicated ultrapure water served as the appropriate vehicle (untreated control). Lipopolysaccharide from *Escherichia coli* O111:B4 (LPS, 10ng/mL, Sigma-Aldrich) or lipoteichoic acid from the cell wall of *Staphylococcus aureus* (LTA, 10ug/mL, Sigma-Aldrich) plus low molecular weight HA, (100ug/ml, as Sodium Hyaluronate, Shandong Topscience Biotech), as well as recombinant human interferon gamma (IFNγ, 50ng/ml, R&D Systems) were prepared following the manufacturer’s instructions and added to culture media when indicated.

### Peripheral blood mononuclear cells isolation

The PBMCs were isolated following a standardized protocol as we previously described (14). Briefly, anti-coagulated peripheral blood samples from healthy volunteers were half diluted (v/v) in PBS and centrifuged at low speed (200 g for 10 min) to separate platelet-rich plasma. The cellular fraction was used to obtain the PBMCs by density gradient centrifugation (400 g for 30 min at room temperature and without brakes) employing Ficoll-Paque Plus (1078 g/ml density, GE Healthcare, Marlborough, MA, USA). The PBMC recovered fraction was washed twice with PBS plus FBS 2%.

All healthy volunteer participants who donated blood samples provided written informed consent. The study was approved by the Institutional Ethics Committee of the National Academy of Medicine, (IMEX-CONICET-ANM) Argentina.

### Monocyte purification and culture

CD14^+^ monocytes were isolated from PBMCs using the EasySep Human CD14 positive selection kit (STEMCELL Technologies, Vancouver, Canada) following the manufacturer’s instructions and as we previously reported (14, 15). Stimulation assays were performed by seeding 2 x 10^5^ isolated CD14^+^ cells in 48-well plates and cultured in complete RPMI-1640 medium (10% FBS and 1% penicillin-streptomycin [PS]) at 37°C and 5% of CO_2_. Inflammatory stimulus (LPS, 10 ng/mL) was added from day 0 in the presence of different concentrations of C8- or C16-C1P (1, 10, and 20 uM) for 24 hours (h) and 48 h. To perform a phospho-protein assay, monocytes were previously serum starved for 3-4 h and then stimulated as indicated. Viability assays were performed by co-staining Annexin V (AnnV) with the fixable viability dye Zombie Violet in monocyte cultures after 24 h of LPS (10 ng/ml) stimulation and different concentrations of C8- and C16-C1P (1, 10, and 20 uM).

### Monocyte-derived macrophage differentiation

As previously described, 2 x 10^5^ CD14^+^ monocytes were plated in 48-well plates containing 500 µL of RPMI 1640 supplemented with 10% FBS and 1% PS plus 50 ng/mL of M-CSF (Immunotools), added at days 0 and 3, to obtain MDM (M0) at day 7 (14, 16). The effect of C8- or C16-C1P on MDM differentiation was carried out by adding 1uM and/or 20 uM of the phospholipids at day 0 and harvesting the cells at day 7. When indicated, apoptotic/necrotic neutrophils or pro-inflammatory stimuli such as LTA and HA or LPS and IFNγ were added to culture media.

To obtain the secretome of each type of MDM, M0 (control), C8- or C16-C1P MDM were boosted with 50nM of Phorbol 12-myristate 13-acetate (PMA) (Sigma-Aldrich) for an additional 4 h on the day 7. These supernatants were collected to use in the angiogenic response assay by analyzing the pseudo-tubule formation.

### Cytokine levels

Supernatants from monocyte cultures were collected, centrifuged, and stored at -80°C until cytokines were measured using commercial ELISA kits (eBioscience, Ready-SET-Go). Pro-inflammatory cytokines (IL-1β, TNFα, and IL-6) were measured at 24 h and IL-10 at 48 h.

### Neutrophil isolation and necrosis induction

Neutrophils were obtained as previously described (17). Briefly, after Ficoll-Paque Plus gradient centrifugation, erythrocytes were sedimented with 6% of dextran (Sigma, St. Louis, MO, USA), and the resulting suspension was collected and centrifuged followed by hypotonic lysis of the remaining erythrocytes. Cells, suspended in RPMI-1640 supplemented with 2% FBS, contained >99.5% neutrophils as determined by May-Grunwald-Giemsa-stained cytopreps. Necrosis was induced by overheating neutrophils suspended in RPMI-1640 supplemented with 2% FBS (10 x10^6^/mL) at 56°C for 10 minutes and then incubating at 37°C and 5% CO_2_ for 18 h prior to co-culture with monocytes. The necrosis/apoptosis ratio was estimated by changes in nuclear morphology and viability labeling with a mixture of acridine orange and ethidium bromide.

### Measurement of angiogenic responses

Using the CD34 positive selection kit (Miltenyi Biotec, Bergisch Gladbach, Germany), endothelial colony forming cells (ECFCs) were isolated from umbilical cord blood collected after normal full-term deliveries with the written informed consent of the mother, as described previously (9, 18). Only ECFCs during the first 40 days of culture were used for experiments. Endothelial cells were cultured in endothelial growth medium 2 (EGM-2, Lonza, Walkersville, MD). A total of 0.15 x 10^6^ cells per well were seeded in 96-well plates with 90% or 50% MDM supernatants diluted with EGM-2. Pseudo-tubule formation on reduced growth factor basement membrane matrix (Geltrex; Gibco, Grand Island, NY) was examined by phase-contrast microscopy, and the total number of branch points was quantified by analyzing images of the entire surface after 18 h of culture. Image analysis was performed with ImageJ.

### Quantitative PCR

For gene expression analysis, MDMs were washed and then harvested with TriZol (Life Technologies, Carlsbad, CA, USA) following the manufacturer’s instructions. Reverse transcription was performed using 100 ng of RNA in 20 μL of reaction volume by employing iScript cDNA synthesis kit (Bio-Rad, Hercules, CA, USA). Real-time PCR reactions were assessed using 1 μL of cDNA in 10 μL of reaction volume by employing SsoAdvanced universal SYBR Green mix and CFX-Connect equipment (Bio-Rad, Hercules, CA, USA). Primers used in this study are listed in **Supplementary S1 Table**. The reaction was normalized to housekeeping gene expression levels, the elongation factor eEF-1 alpha (EEF1A1), and the specificity of the amplified products was checked through analysis of dissociation curves.

### Surface and intracellular staining and flow cytometry

The activation markers of monocytes and polarizing phenotype of macrophages were characterized by cell surface staining employing the appropriate combination of directly conjugated antibodies against human CD11b-APC/Cy7 (BioLegend, RRID: AB_830641), CD64-APC (BioLegend, RRID: AB_1595539), CD163-PerCP/Cy5.5 (BioLegend, RRID: AB_2650629), CD206-AlexaFluor 488 (BioLegend, RRID: AB_571874), CD14-PECy7 (BioLegend, RRID: AB_830691), HLA-DR-FITC (BioLegend, RRID: AB_2616625), CD80-PE (BioLegend, RRID: AB_314503), and PECy7-CD44 (Biolegend, RRID: AB_830786) following standard protocol. Briefly, the harvested cells were washed with 1x PBS and blocked in 1x PBS 5% FBS at room temperature for 15 min. The cells were washed with 1x PBS and the respective antibody cocktails (prepared in 1x PBS/2% FBS) were added to the cell pellet and incubated at 4°C for 30 min. In viability assays, FITC-Annexin V (BioLegend RRID: AB_2561292) was incubated for 15min at room temperature in binding buffer. A fixable viability dye Zombie Violet (BioLegend) was used according to the manufacturer’s instructions to gate on live cells or to identify apoptotic cells in combination with Annexin V. After washing, cells were fixed with a Cytofix/Cytoperm Kit (BD Biosciences) and washed again.

For intracellular staining, after fixation, cells were permeabilized using Perm/Wash Buffer I (BD Biosciences) and then cells were incubated with FITC-anti-BCL2 antibody (Ebioscience, RRID: AB_10596978) at 4°C for 30 min, and finally washed. For phosphoflow experiments, after stimulation, cells were fixed and permeabilized with Perm III solution (BD Biosciences). FITC phospho-ERK1/2 (Biolegend, RRID: AB_2721568) was employed for intracellular staining. Cells were acquired using a FACS Canto I cytometer (Becton Dickinson), or a Sysmex CyFlow Partec, and all analysis was carried out with FlowJo software (Tree Star). The negative control fluorescence minus one (FMO) was used to set a negative signal in interested channels. The technical information of each antibody is listed in the **Supplementary S2 Table**.

### Comparison of C8-C1P-induced MDM gene profile in the MoMacverse framework

The MoMacverse is a publicly available tool to map specialized cell subsets of monocytes and macrophages widely distributed across multiple tissues and defined by conserved gene signatures in health and disease. We first performed a mean normalization. The gene means across all conditions were computed for control. Then, each condition of C8-C1P MDM was normalized to the mean of the control MDM. As a result, if the point value is less than the mean, it will have a negative sign; otherwise, it will be positive; furthermore, the mean division allowed us to compare different genes from different experiments. Finally, the mean for each gene and condition was computed; if this mean resulted in a negative value, it was considered a zero expression. This strategy results in the following gene expression sets; C8_20uM: VEGFA, MER (MERTK), PPARG, LXRA (NR1H3), IL10, FOXO1, and FGF2. C8_1uM: VEGFA, MER (MERTK), PPARG, LXRA (NR1H3), IL10, CD163 and MRC1. C8_1 + 20uM: VEGFA, MER (MERTK), PPARG, LXRA (NR1H3), IL10, PDGFA, FGF2, CD163 and MRC1. Control: IL4, FOXO1, GAS6, CD36, IRF1, TYRO3, TGFB1, PDGFA, and IGF1. We then used these gene sets to load into the app and compared the C8-C1P-induced MDM with the MoMacverse framework https://macroverse.gustaveroussy.fr/2021_MoMac_VERSE/.

### Statistical analysis

Data are expressed as mean ± SEM. The Shapiro-Wilk test was used to define state normality and equal variance. Significant differences were determined by one-way analysis of variance (ANOVA) followed by Dunnett´s (in case of testing several concentrations vs a reference control) or Fisher multiple comparison test. All statistical descriptions used in each experiment are described in the respective figure legends. A minimum of 4 and a maximum of 7 donors were employed for every experiment. Statistical significance was set at p <0.05. The analysis was performed using GraphPad Prism software.

In order to identify hidden patterns in a data set, reduce its dimensionality by removing the noise and redundancy, and identify correlated variables, Principal Component Analysis (PCA) was computed in RStudio 1.4.1106 using missMDA, FactoMineR, FactorExtra, Ggplot2, and Corrplot packages. First NA missing values were imputed and continuous variables were detected automatically. Eigenvalues were then visualized, and the percentage of variances was explained by each principal component. Graphics and clustering analysis were performed using FactorExtra, Ggplot2, and Corrplot packages.

## Results

### Synthetic C8-C1P but not natural C16-C1P dampen inflammatory markers in LPS-challenged monocytes

**Figure 1:**
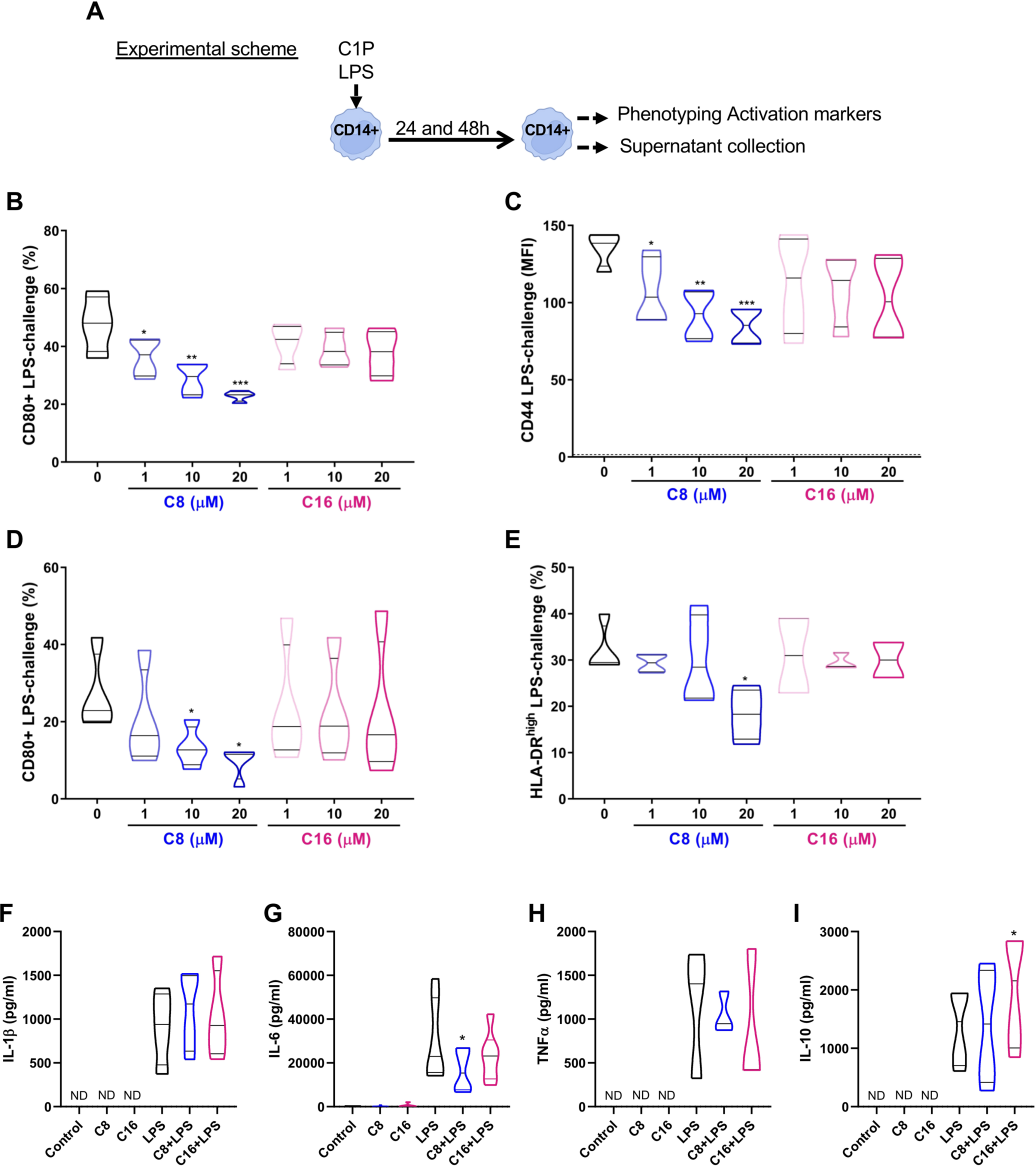
C1P modulated pro-inflammatory molecules and soluble mediators in LPS-challenged human CD14 monocytes. **(A)** Experimental scheme and **(B, C)** Quantification of CD80^+^ cells and CD44 level (indicated as % and MFI, respectively) on isolated CD14^+^ monocytes challenged with 10 ng/ml of LPS for 24 h, and in the absence or presence of different concentrations of C8-C1P or C16-C1P (1-20 µM). **(D, E)** Percentage of CD80^+^ and HLA-DR^high^ monocytes after 48 h of LPS challenge (10 ng/mL) in the absence or presence of different concentrations of C8-C1P or C16-C1P (1-20 µM). **(F-I)** Cytokine production in the culture supernatant of CD14^+^ monocytes challenged with 10 ng/mL of LPS in the absence of presence of 20µM of C8-C1P or C16-C1P. Pro-inflammatory cytokines, IL-1β, IL-6 and TNFα, were measured at 24 h while the anti-inflammatory cytokine IL-10 was measured at 48 h. One-way paired ANOVA and Dunnett’s *post hoc* test, statistical significances *p<0.05, **p ≤ 0.01, ***p<0.005, ****p ≤ 0.001. Violin plots show the median and quartiles. Independent data from each experiment, n = 4–7.

Considering the relevance of circulating monocytes as infiltrating cells and precursors of macrophages in the injured tissue, we first aimed to evaluate the specific effect of C1P treatment on human-isolated CD14^+^ monocytes (Experimental scheme **Figure 1A**). Interestingly, we found that treatment with C8-C1P (**Figure 1B**) but not C16-C1P (**Figure 1C**) decreased the expression of CD80 and CD44 in a concentration-dependent manner (1-20 µM) on LPS-challenged monocytes (10ng/mL), after 24 h. A similar effect was observed on the CD80 and HLA-DR molecules after 48 h of LPS challenge (**Figures 1D, E**). Representative dot plots for the activation markers are shown in **Supplementary Figure S1A**. The highest concentration of both ceramide C1P (20µM) did not induce cytokine release by itself (**Figures 1F–I**), but it was able to dampen IL-6 production induced by LPS stimulation after 24 h (**Figure 1G**). No changes were observed in other classical pro-inflammatory cytokines, such as IL-1β and TNFα (**Figures 1F, H**, respectively), indicating that the phospho-sphingolipid does not affect the ability of monocytes to secrete these cytokines when activated by bacterial products, a critical feature for pathogen protection in wounds. The anti-inflammatory cytokine IL-10 peaked later and it was measured at 48 h of LPS challenge, with a significant increase observed only in the presence of the ceramide C16-C1P (**Figure 1I**).

To analyze if the phospho-sphingolipid C1P affects monocyte survival at the concentration of 0–20µM, a viability assay using Annexin V (AnnV) and the fixable viability dye Zombie Violet was performed. We observed that both C16-C1P and C8-C1P significantly reduced the percentage of early apoptotic monocytes (AnnV^+^ Zombie Violet^-^) at 20 µM (**Supplementary Figure S1B**) after 24 h. We also observed a significant increase of BCL-2 at 24 h and 48 h in monocytes treated with C8-C1P compared with non-treated controls (**Supplementary Figure S1C**). In concordance with BCL-2, human CD14^+^ monocytes exposure to C8-C1P induced a significant increase of phosphorylated ERK1/2 (p-ERK1/2) at 20µM after 15 or 30 minutes compared with non-stimulated cells (**Supplementary Figure S1D**).

### Primed C8-C1P monocytes augmented pro-angiogenic properties of macrophages´ secretome and skewed differentiation toward the pro-resolutive phenotype

As previously described, C1P is a chemoattractant molecule for murine macrophages and other murine and human progenitor cells (9). To test whether our synthetic ceramide C8-C1P has the ability to chemoattract human monocytes, a transwell migration assay was performed. Purified CD14^+^ monocytes were seeded in the upper chamber while media with 1µM of C8-C1P, a low concentration mimicking a distal signal from the injured tissue, was placed in the bottom chamber. The phospholipid C8-C1P significantly increased the migration of monocytes through the transwell membrane at 1µM compared with the medium without the phospholipid. Additionally, the supernatant of hECFCs was also able to induce migration of monocytes that was enhanced when hECFCs were treated with 1µM of C8-C1P, showing a direct and indirect effect of the phospholipid on monocyte recruitment (**Supplementary Figure S2A**).

**Figure 2:**
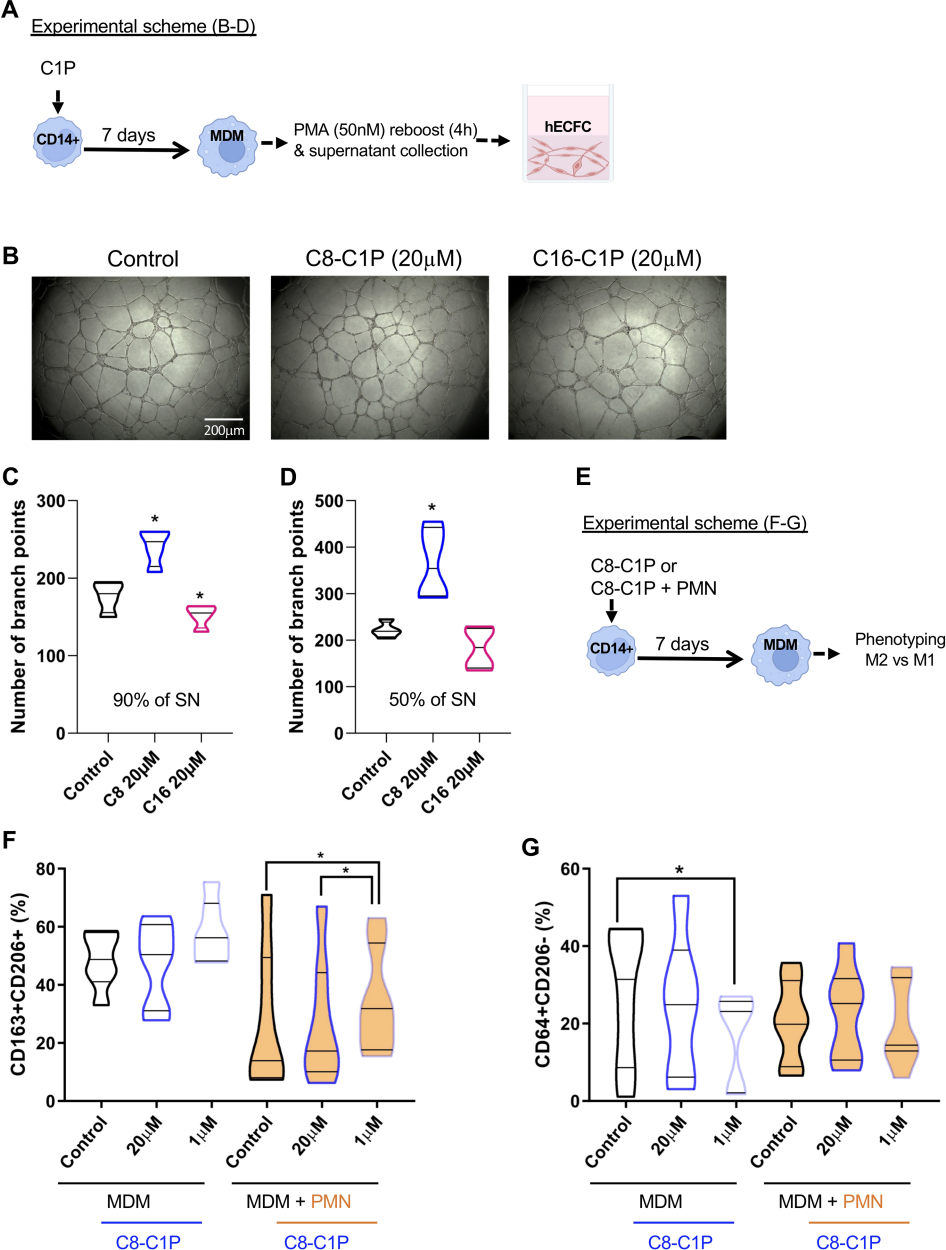
C8-C1P priming conferred pro-angiogenic properties to MDM secretome with high doses and increased M2 markers with low doses. **(A)** Experimental scheme and **(B)** representative photographs of capillary-like tubule formation after 18hs of *in-vitro* assay using human ECFC progenitors. The supernatant (SN) of C1P-MDM (C8-, C16-) or control MDM were collected at day 7 after reboost with PMA (50nM) in the last 4h. **(C, D)** Number of branch points counted in the capillary-like tubule formation assay after 18hs of culture. hECFCs were cultured with 90% in C, and 50% in D of SN of C1P-MDM. **(E)** Experimental scheme and **(F, G)** Percentage of M2 (CD206^+^CD163^+^) or M1 (CD64^+^CD206^-^) markers on control MDM and C8-C1P-MDM (**F, G** respectively). A pro-inflammatory stimulus employing necrotic neutrophils (PMN) were also included in the MDM differentiation and the percentage of M2 (CD206^+^CD163^+^) or M1 (CD64^+^CD206^-^) markers were compared with its respective control. MDM = monocyte-derived macrophages. One-way paired ANOVA and Fisher’s *post hoc* test, statistical significances *p<0.05. Violin plots show the median and quartiles. Independent data from each experiment, n = 4–7.

After inflammation, the crosstalk of macrophages and ECFCs is central for the re-vascularization process of the injured tissue. Thus, we tested if MDM differentiated from C1P-primed monocytes (C1P-induced MDM) were able to increase the pro-angiogenic properties of hECFCs. A tubulogenesis assay with hECFCs was performed employing the supernatant (secretome) of day 7 C1P-induced MDM, reboosted with 50nM of PMA for the last 4 h. The experimental scheme is shown in **Figure 2A**, and representative photographs of capillary-like tubule formation in **Figure 2B**. Interestingly, C8-C1P (20µM), but not C16-C1P, significantly increased the angiogenic properties of the C8-C1P-induced MDM secretome, when used at 90%, by augmenting the branch points formation of hECFCs (**Figure 2C**). Moreover, a higher number of branch points was observed when supernatants were diluted in half (50%) (**Figure 2D**), which could be due to the dilution of pro- and anti-angiogenic factors. Considering C8-C1P promoted the most significant changes, we continued our next experiments using only this ceramide. The pro-angiogenic property of the C8-C1P-induced MDM secretome is mainly observed in the presence of higher concentrations (20µM) (**Supplementary Figure S2B**).

To mimic the injured tissue, we differentiated C8-C1P-induced MDM in the presence or absence of necrotic neutrophils, which represent an inflammatory environment (experimental scheme, **Figure 2E**). We observed that priming monocytes with only 1µM of C8-C1P promotes MDM expressing M2-like markers (CD163^+^CD206^+^) as shown in **Figure 2F**. The presence of necrotic inflammatory stimulus reduced the M2 markers already in the control condition, but the presence of C8-C1P treatment significantly increased CD163^+^CD206^+^ markers compared with its respective control. The classic M1 phenotype, defined by CD64^+^CD206^-^ cells, was significantly reduced in C8-C1P-induced MDM with 1µM compared with control MDM (**Figure 2G**). The gating strategy is shown in **Supplementary Figure S2C**

### C8-C1P-induced MDM display an anti-inflammatory and pro-angiogenic program compared with control macrophages

**Figure 3:**
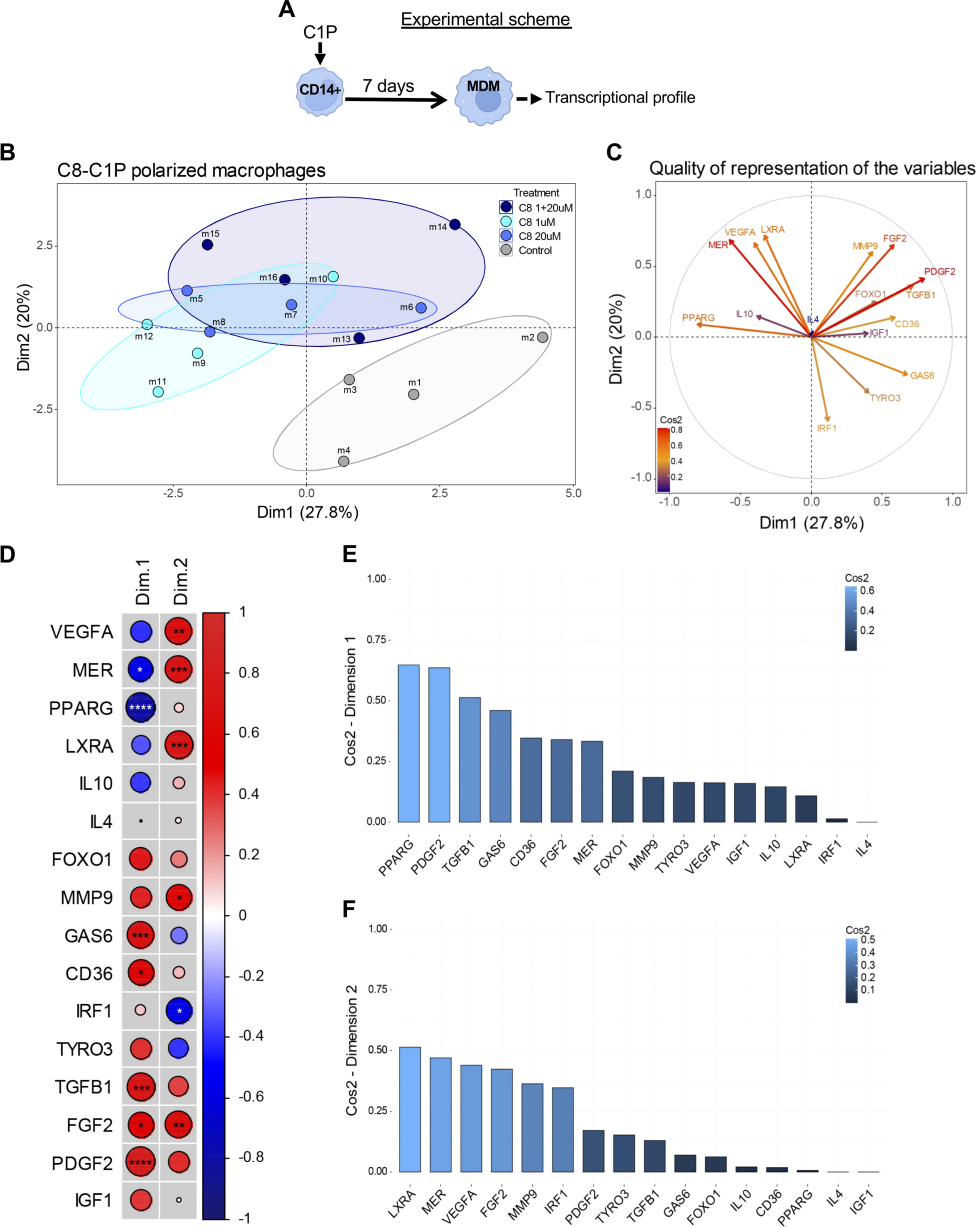
Macrophages derived from C8-C1P-primed monocytes display an anti-inflammatory and pro-angiogenic program compared with control macrophages. **(A)** Experimental scheme and **(B)** Individual Factor Map showing spatial distribution of different conditions of MDM colored by group, control MDM in gray, C8-C1P-induced MDM from 1µM in turquoise, from 20µM in blue, and from the sequential combination of both (1 + 20µM) in dark blue). **(C)** Circle of correlation with vectorized variables (expression parameters) showing their interrelationship and colored by Cos^2^ values. **(D)** Correlation matrix among all gene analyzed in Dimensions 1 and 2. Positive correlation is indicated in red and negative correlation in blue and significant changes denoted with asterisk. **(E, F)** Weight of individual variable contributions listed by their Cos^2^ values for Dimension 1 **(E)** and Dimension 2 **(F)**, respectively. MDM = monocyte-derived macrophages. In **(D)** Pearson correlation coefficient was used; statistical significances *p<0.05, **p ≤ 0.01, ***p<0.005, ****p ≤ 0.001. Independent data from each experiment, n = 4.

To characterize the gene program induced by C8-C1P on MDM, a principal component analysis (PCA) was carried out based on the expression of 16 genes involved in lipid metabolism, anti-inflammatory response, angiogenesis, and survival by qPCR (experimental scheme, **Figure 3A**). The Individual Factor Map (**Figure 3B**) clearly shows that control macrophages (in gray) differentially segregate from C8-C1P-induced MDM (1µM in turquoise, 20µM in blue, and the sequential combination of both in dark blue) based mainly on dimension 1. These results indicate that C8-C1P-treated MDM expresses a differential transcriptional profile compared with non-treated MDM. The first two dimensions explain 47.8% of the total variability contained in the samples. **Figure 3C** shows the vectorized correlation and **Figure 3D** is a correlation matrix among all variables analyzed in the two first spatial dimensions. Thus, genes such as PPARG and MERTK negatively correlate with PDGFB, TGFB1, GAS6, or CD36 in Dimension 1 (**Figures 3C, D**). Similarly, a positive correlation was seen for anti-inflammatory LXR-alpha and MERTK with pro-angiogenic genes VEGFA, FGF2, and Metallopeptidase-9 in dimension 2 (**Figures 3C, D**). The IRF1 gene, a characteristic M1-like macrophage transcription factor, is inversely correlated with the anti-inflammatory or pro-angiogenic genes (**Figures 3C, D**). The weight or individual contributions of each variable for Dim 1 and Dim 2 are shown in **Figures 3E, F**.

To understand where C-8-C1P-induced MDM localize within the cross-tissue landscape of human monocytes and macrophages in health and disease, we compared them with the online-available platform MoMacverse (19). After gene normalization, we compared the gene expressions of our 4 MDM conditions (Control, C8_1uM, C8_20uM, and C8_1 + 20uM) under the MoMacverse framework. The gene expression profile of each condition and the representative maps for C8-induced MDM, showing those cells that most strongly express their respective gene signatures, are visualized in **Supplementary Figure S3A-D**. The blue points indicate where the genes are expressed. The specific localization into the map is related to a given lineage in the MoMacverse (**Supplementary Figure S3E**). Even though we only analyzed a few genes, it is noteworthy that this differential gene expression of C8-C1P-induced MDM locates these macrophages around the HES1-Macrophage-2 and Macrophage cluster-7 of MoMacverse, Mulder et al. (19), as is shown in **Supplementary Figure S3B-D**.

### A wound inflammatory environment, driven by LTA and HA, promotes a strong M1 program that is restrained by C8-C1P

**Figure 4:**
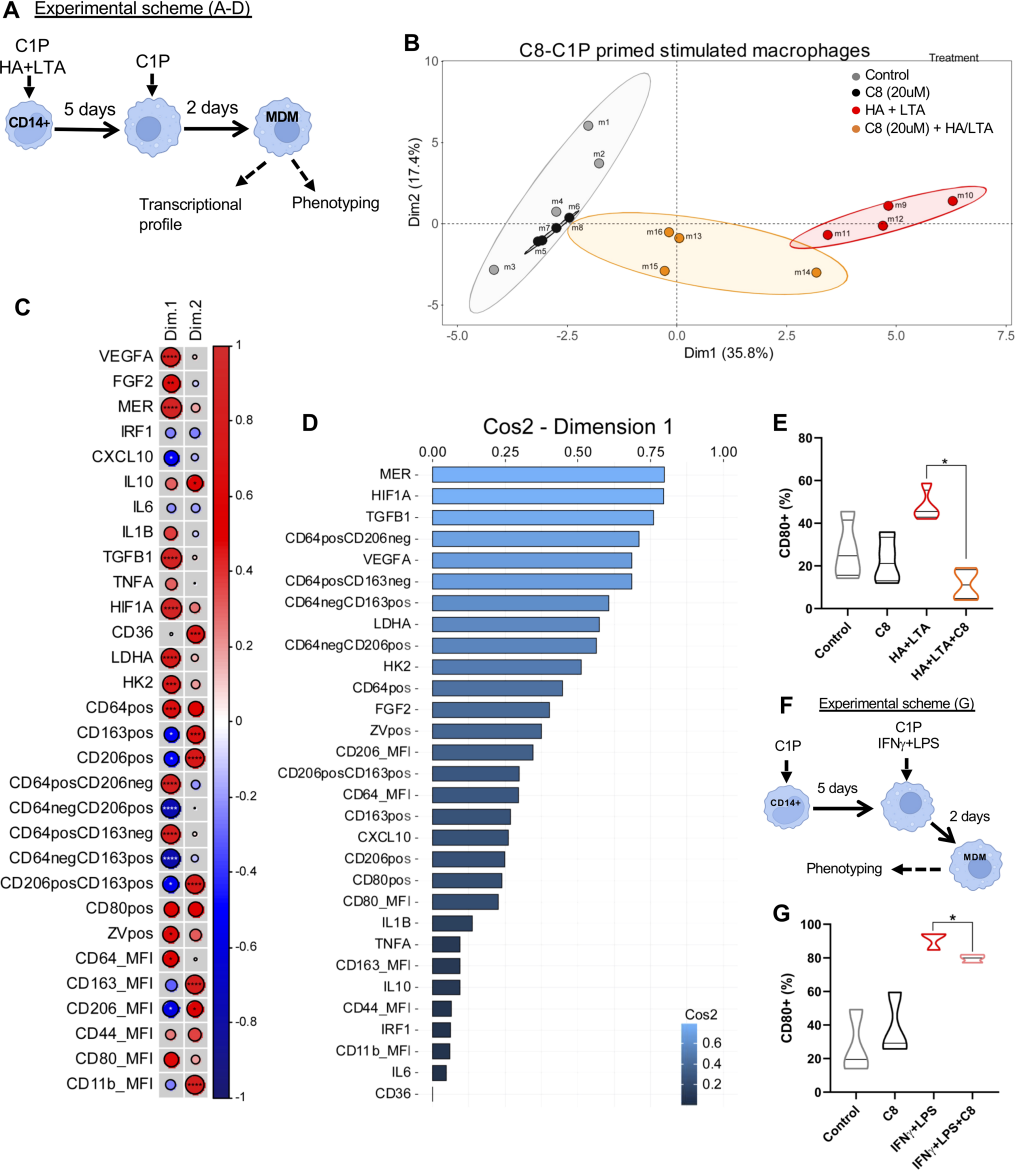
C8-C1P restrains pro-inflammatory M1 program induced by LTA and HA in MDM. **(A)** Experimental scheme and **(B)** Individual Factor Map showing how each type of MDM distribute separately in the space according to Dimension 1 and 2, (> 50% of variance). Each group is indicated with a different color, control and C8-C1P-MDM in gray and black dots respectively, LTA-HA-MDM identified with red dots and the combination of LTA-HA-MDM with C8-C1P in orange. **(C)** Correlation matrix of variables and Dimensions 1 and 2 showing those variables with significant changes. Positive correlation is indicated in red and negative correlation in blue and significant changes denoted with asterisk **(D)** Most contributing variables listed by their Cos^2^ values for Dimension 1. **(E)** CD80 expression in MDM differentiated in the presence of pro-inflammatory LTA (20ug/mL) plus HA (100ug/mL), and in the presence or absence of C8-C1P (20uM). **(F)** Experimental scheme and **(G)** CD80 expression in C8-C1P-MDM treated with canonical M1 skewing stimulus IFNγ (50ng/mL) plus LPS (10ng/mL). C8-C1P used at 20uM. LTA = Lipoteichoic acid, HA = low molecular weight hyaluronic acid. In **(C)** Pearson correlation coefficient was used; in **(E, G)** one-way paired ANOVA and Fisher’s *post hoc* test was used; statistical significances *p<0.05, **p ≤ 0.01, ***p<0.005, ****p ≤ 0.001. Independent data from each experiment, n = 4.

In order to mimic a wound inflammatory milieu, MDM was also differentiated in the presence of lipoteichoic acid (LTA) plus low molecular weight hyaluronic acid (HA), as well as in combination with C8-C1P 20µM (experimental scheme, **Figure 4A**). LTA is a PAMP from the cell wall of *Staphylococcus aureus*, the most common opportunistic commensal bacteria found in exposed chronic wounds while low molecular weight HA is a common inflammatory DAMP derived from the extracellular matrix rupture upon physical or biochemical damage. A new PCA was performed combining gene expression profile (14 genes by qPCR) with phenotypic markers (16 variables obtained by flow cytometry). The first two dimensions explained more than 53.2% of the total variability contained in our pooled samples. Dimension 1 segregated MDM based on their inflammatory status promoted by LTA and HA (**Figure 4B**). Thus the inflammatory MDM (LTA and HA) identified with red dots clearly clustered separately from the control and C8-C1P-MDM (gray and black dots). Interestingly, inflammatory MDM combined with C8-C1P (orange dots) restrains the acquisition of the inflammatory program (**Figure 4B**). A correlation circle containing vectors that display the existing relationships among pro- and anti-inflammatory, growth factor, and angiogenic gene expression with the phenotypic macrophage markers and their corresponding dimensions are shown in **Supplementary Figure S4A**.

The correlation matrix (**Figure 4C**) denoted a strong correlation among inflammatory variables such as CD64 and pro-glycolytic genes (HIF1A, HK2, and LDHA), but also with anti-inflammatory, growth factor, and angiogenic genes (MERTK, TGFB1, FGF2, and VEGFA) (**Figure 4C**). This is consistent with the inverse correlation with CD206 and CD163 expression (prototypical M2 markers) and CXCL10 expression across Dimension 1 (**Figure 4C**). The weight or individual contributions of each variable for Dim 1 is shown in **Figure 4D**. Altogether, the C8-C1P treatment during inflammatory MDM differentiation restrains the inflammatory M1 program and favors pro-resolutive or tissue repair programs. Furthermore, C8-C1P-primed MDM was protected from cell death under the LTA and HA inflammatory milieu (**Supplementary Figure S4B**), as we also observed in LPS challenge CD14 monocytes (**Supplementary Figure S1B, C**). Finally and as a proof of concept that priming monocytes with C8-C1P reduce polarization to M1, we show that macrophages differentiated under LTA and HA inflammatory milieu or under the canonical M1 skewing condition (LPS + IFNγ) but in the presence of C8-C1P significantly reduced the level of CD80 (**Figures 4E–G** respectively).

## Discussion

Chronic wounds represent a major and increasing health and economic burden in modern societies. It often occurs in patients with one or several underlying disorders, e.g., venous or arterial insufficiency, diabetes mellitus, or systemic inflammatory disease, trapping wounds in a constant inflammatory state and failing to progress through the normal healing stages. Chronic wounds are difficult to treat due to their complex nature and our limited understanding of pathogenetic mechanisms. Recurrence is also a common episode in treated patients and to date, no satisfactory and affordable treatments are available; therefore, new therapeutic targets are urgently needed (20–22).

Currently, both pro- and anti-inflammatory properties of the phospho-sphingolipid C1P have been reported, and which results depend on the cellular compartment of production and signaling (10, 23–27). Here we have shown that C8-C1P but not C16-C1P treatment was able to reduce pro-inflammatory markers (CD44, CD80, and HLA-DR in addition to IL-6 secretion) in human-isolated CD14^+^ monocytes challenged with LPS. These results indicate different immunoregulatory properties of both forms of C1P, a key aspect considering that C16-C1P is the natural form and C8-C1P could be exogenously administered. Previous work from Hankins et al (25) showed that C8-C1P selectively blocks the TLR4-NFkB axis reducing MAPK activation and cytokines expression, which is in concordance with our observation of CD44, CD80, HLA-DR, and IL-6 downregulation by monocytes after LPS challenge. An interesting observation was that C8-C1P treatment does not completely ablate the capacity of monocytes to secrete pro-inflammatory cytokines such as TNFα or IL-1β at 24 h after LPS challenge, a critical aspect for pathogen control. These results suggest that C8-C1P is not a pleiotropic inhibitor, and additional research is needed to determine the specific mechanism of IL-6 downregulation by C8-C1P in monocytes. In previous reports, it has been shown that C8-C1P was able to inhibit TNFα production only when J774 cells were treated with low LPS concentrations (1ng/mL) but not at higher LPS concentrations (100 ng/mL) (24, 25). Additionally, Al-Rashed et al. showed that cytoplasmic C1P, released by endogenous metabolism *via* CERK, induces opposite effects to outside-cell C1P (exogenous administration) in response to the stimulation with TNFα, suggesting different effector targets and mechanisms of C1P (27).

A well-reported property of C1P is its ability to induce cell survival, counteract apoptotic stressors, and induce proliferation in different models (8, 28, 29). Here we showed that both types of phospholipids, the natural C16-C1P and the synthetic C8-C1P, promote antiapoptotic activity after serum deprivation in freshly isolated human CD14^+^ monocytes. Moreover, the anti-apoptotic pathway induced by C8-C1P involved the up-regulation of BCL-2 and ERK 1/2 signaling. These results are in concordance with previous reports showing that natural C16-C1P is able to up-regulate another BCL-2 subfamily member – BCL-XL – in apoptotic macrophages (30), and that the phospho-sphingolipid C1P triggers ERK and AKT signaling pathways in several cell types (9, 29, 31, 32). Additionally, Jung et al. showed that C2, C6, and C8 ceramides as well as C8 ceramide-1-phosphate inhibited iNOS and proinflammatory cytokines in LPS-stimulated BV2 microglial cells and rat primary microglia.

Taking into account that monocytes are very plastic cells, we certainly considered that priming monocytes with C8-C1P would affect the differentiation and polarization program of MDM and consequently their effector response. We found that C8-C1P but not C16-C1P was able to instruct MDM to secrete more pro-angiogenic factors, which was enhanced when the supernatant was diluted in half, suggesting a balance between anti and pro-angiogenic factors in the C8-C1P-MDM secretome. Previously, using a model of hECFCs, we found that both C8-C1P and C16-C1P treatments were able to increase angiogenesis properties, but the synthetic form was more powerful. The concentration of C8-C1P is another critical aspect depending on the cell type since 10µM of C8-C1P is already toxic for hECFCs (9), while for monocytes higher concentrations are well tolerated and even protective. These results exemplify how different forms and concentrations of C1P could have a different impact depending on the cell type and tissue circumstances.

During wounding, macrophages do not exhibit discreet and static phenotypes; they instead harbor a complex and dynamic combination of markers, resembling inflammatory (also called M1) but also alternatively activated macrophages (a broader spectrum of M2) (3, 33). Here we aimed to characterize C8-C1P-MDM in the presence of necrotic cells, which are known to be a common feature of chronic and poorly irrigated injured tissues (9, 34). Priming monocytes with low C8-C1P concentration (1μM) increased CD206^+^ and CD163^+^ M2 markers and reduced CD64^high^ expression, a classical M1 marker. The same result was observed even in the presence of necrotic cells, mimicking a pro-inflammatory environment. Furthermore, the PCA analysis supports that C8-C1P is indeed promoting the induction of anti-inflammatory and pro-angiogenic genes compared to the control condition. PPARγ, MER, and LXRα are known to be induced by the lipid content of apoptotic-engulfed corpses or by anti-inflammatory lipid mediators and to integrate lipid metabolism with anti-inflammatory mechanisms (35–37). In particular, PPARγ not only operates as a ligand-dependent nuclear receptor, but is also a molecular signature of alternatively activated macrophages (38–40). The pro-proliferative and pro-angiogenic factors like FGF2, VEGFα, and MMP9 are in concordance with the tubulogenic assay and could be mediating the increased tubulogenesis in human endothelial progenitors. In these cells, C8-C1P also dampens the classical M1 transcription factor IRF1 during differentiation.

The MoMacverse provides unified and conserved gene signatures in an array of specialized monocyte and macrophage subsets widely distributed across multiple human tissues in health and disease (19). Taking advantage of this robust online-available platform, we tested where the C8-C1P-induced MDM localizes in the specific macrophage map. Even though we have a low number of genes mainly focused on pro-angiogenic and pro-resolutive transcription programs, the C8-C1P-MDM matches the region of HES1-Macrophage-2 and Macrophage cluster-7 of MoMacverse (19). The HES1-Macrophage-2 is characterized by the expression of FOLR2 and CD206 (MRC1) receptors as well as an M2 signature, and the differentially expressed regulons (DERs) analysis showed that this macrophage also shared a similar EDR profile with TREM2_Mac (#3) (19). Nonetheless, given the limitations of our data and considering the low number of genes analyzed, it will be fundamental for more research to further support our premises, and also produce *in-vivo* models to define their effector response.

Chronic wounds are usually “stalled” in the inflammatory phase without an active transition from the M1 to an M2-like phenotype, a step required for the tissue repair and remodeling process. Such pathological circumstance is characterized by waves of inflammatory leukocytes, activation of proteolytic enzymes, cell death, ROS production, pro (and anti) -inflammatory cytokines, and arachidonic acid-derived metabolites (41, 42). In order to resemble the complex microenvironment of a chronic wound, we differentiated MDM in the presence of two common inflamogens, LTA from *Streptococcus aureus* and low molecular weight HA. Our results showed that LTA-HA-MDM are segregated apart with an inflammatory phenotype compared to those cells that were treated with C8-C1P. Furthermore, C8-C1P restrained the acquisition of inflammatory programs despite the presence of LTA and HA. Persistent inflammation biases MDM to an M1-program which contributes to fibroblasts and pericytes activation, proliferation, and differentiation into ECM-producing myofibroblasts in a variety of fibrotic diseases, including hypertrophic scars and keloids (33, 43–45). Therefore, efficient control of pro-inflammatory macrophages is critical to minimizing scar formation and preventing excessive scarring.

The emerging picture of tissue macrophages derived from circulating monocytes is the feature in response to a loss of homeostasis, in which they participate in the induction and resolution of inflammation by moving along defined activation paths. The commitment of monocytes to these activation paths is regulated not only by the inflammatory settings but also by the specific nature of the tissue. It is possible that through phagocytosis, macrophages become tissue imprinted that convey tissue identity (46). Phagocytic and inflammatory paths are the most common gene expression programs that are responsible for shaping macrophage function (46, 47).

This study also showed that after sustained inflammatory conditions, C8-C1P could protect MDM from cell death and prevented CD80 up-regulation, suggesting that this lipid has long-term effects on the macrophages differentiated from primed monocytes. This result could be explained by the new concept of trained immunity, given that C8-C1P-primed MDM harbor distinctive transcriptional profile features. In recent years, evidence has accumulated supporting the idea that innate immune cells, when exposed to a primary PAMP or DAMP, undergo physiological and epigenetic adaptations that will ultimately allow the same cells to respond quickly or even more effectively to secondary homo or heterotypic challenges (48). In this particular case and as far as we explored, imprinting triggered by C8-C1P conditioned MDM protects them from cell death under prolonged inflammatory conditions, reducing co-stimulation, and skewing the gene profile in favor of resolution and tissue repair functions.

The synthetic sphingophospholipid, C8-C1P, has demonstrated extensive therapeutic potential, particularly in some pathological scenarios such as non-allergic asthma, smoke-induced airway inflammation, chronic diabetic wounds, ischemia, and other conditions involving injured tissues and a proinflammatory background (12). One of the more remarkable advantages is the lipidic nature of C8-C1P which contributes to its chemical stability and provides versatility for distinct possible pharmacological presentations (eg, inhalable aerosols, injectable emulsions, pills, ointments, and creams, among many others) (10, 11, 26, 49). However, our knowledge about C1P remains limited and many questions are still unanswered regarding the mechanistic action and cellular response to this phospholipid.

## Data availability statement

The original contributions presented in the study are included in the article/**Supplementary Material**. Further inquiries can be directed to the corresponding author.

## Ethics statement

The studies involving human participants were reviewed and approved by Institutional Ethics Committee of the National Academy of Medicine (IMEX-ANM). The patients/participants provided their written informed consent to participate in this study.

## Author contributions

JMOW and HAM: Data curation, Formal analysis, Investigation, Methodology, Validation, Visualization, Writing-original draft, Writing-review, and Editing. CMO: Data curation, Formal analysis, Investigation, Methodology, Software, Validation, Visualization, Writing-review, and Editing. MML: Data curation, Formal analysis, Software, Validation, review, and Editing manuscript. EP: Investigation, Methodology, Software, Validation, and Visualization. MS and SN: Conceptualization, Methodology, Resources, Writing-review, and Editing. AEE: Conceptualization, Funding acquisition, Methodology, Supervision, Resources, Writing-review, and Editing. EACS: Conceptualization, Funding acquisition, Methodology, Supervision, Project administration, Funding acquisition, Resources, Writing-original draft, Writing-review, and Editing. All authors contributed to the article and approved the submitted version.
