# Supplementary material for: The synthetic phospholipid C8-C1P determines pro-angiogenic and pro-reparative features in human macrophages restraining the proinflammatory M1-like phenotype
Source: Front Immunol. 2023 Jun 16;14:1162671. doi: 10.3389/fimmu.2023.1162671 (PMC10311553; doi:10.3389/fimmu.2023.1162671)
Supplement: Supplementary file 1 [file Presentation_1.pdf]

Supplementary Material

The synthetic phospholipid C8-C1P determines pro-angiogenic and pro-reparative features in human macrophages restraining the proinflammatory M1-like phenotype.

Juan Manuel Ortiz Wilczyñski [1], Hebe Agustina Mena [1, †], Martin Ledesma[1], Cinthia Mariel Olexen [1,2], Enrique Podaza [†], Mirta Schattner [1], Soledad Negrotto [1], Andrea Emilse Errasti [2], Eugenio Antonio Carrera Silva [1*]

* **Correspondence**: Eugenio Antonio Carrera Silva, Pacheco de Melo 3081, Ciudad Autónoma de Buenos Aires, 1425, (+54) 011 48091048, carrerasilva@yahoo.com.ar.

1 Supplementary Figures and Tables

This file includes

Table S1-S2.

Figure S1, S2, S3 and S4.

1.1 Supplementary Tables

Supplementary S1 Table: List of Primers sequences**.**

Oligo name	Sequence
hCD36 Fwd	TCTTTCCTGCAGCCCAATG
hCD36 Rv	AGCCTCTGTTCCAACTGATAGTGA
hCXCL10 Fwd	AGCAGTTAGCAAGGAAAGGTCTA
hCXCL10 Rv	TGAAGCAGGGTCAGAACATCC
hEEF1A1 Fwd	TCGGGCAAGTCCACCACTAC
hEEF1A1 Rv	CCAAGACCCAGGCATACTTGA
hFGF2 Fwd	CCAGCAGAAAGAGGAAAGAGGTAG
hFGF2 Rv	CCCCAAAAGCAGGTCACTCAC
hFOXO1 Rv	GTGTAACCTGCTCACTAACCC
hFOXO1Fwd	ATGTGTTGCCCAACCAAAGC
hGAS6 Fwd	GGCAGACAATCTCTGTTGAGG
hGAS6 Rv	GACAGCATCCCTGTTGACCTT
hHIF1A Fwd	ACTAGCCGAGGAAGAACTATGAA
hHIF1A Rv	TACCCACACTGAGGTTGGTTA
hHK2 Fwd	GAGCCACCACTCACCCTACT
hHK2 Rv	CCAGGCATTCGGCAATGTG
hIGF1 Fwd	AGGAAGTACATTTGAAGAACGCAA GT

hIGF1 Rv	CCTGCGGTGGCATGTCA
hIL10 Fwd	GCCGTGGAGCAGGTGAAG
hIL10 Rv	GAAGATGTCAAACTCACTCATGGCT
hIL1B Fwd	ACCAAACCTCTTCGAGGCAC
hIL1B Rv	ATCGTGCACATAAGCCTCGT
hIL4 Fw	CCAACTGCTTCCCCCTCTG
hIL4 Rv	TCTGTTACGGTCAACTCGGTG
hIL6 Fwd	TTCGGTACATCCTCGACGGC
hIL6 Rv	TCACCAGGCAAGTCTCCTCA
hIRF1 Fwd	CACTAACATTTCCCCCGAGC
hIRF1 Rv	ATGTCCCTGTTCACCCCAAAG
hLDHA Fwd	TGGGAGTTCACCCATTAAGC
hLDHA Rv	AGCACTCTCAACCACCTGCT
hMER Fwd	CTCTGGCGTAGAGCTATCACT
hMER Rv	AGGCTGGGTTGGTGAAAACA
hMMP9 Fwd	TCTATGGTCCTCGCCCTGAA
hMMP9 Rv	TCGCCAGTACTTCCCATCCT
hNR1H3 Fwd	GGAGGTACAACCCTGGGAGT
hNR1H3 Rv	AGCAATGAGCAAGGCAAACT
hPDGFB Fwd	CCATTCCCGAGGAGCTTTATG

hPDGFB Rv	CAGCAGGCGTTGGAGATCAT
hPPARG Fwd	GACCACTCCCACTCCTTT
hPPARG Rv	CGACATTCAATTGCCATGAG
hRAR alpha Fwd	GCCTGGACATCCTGATCCTG
hRAR alpha Rv	TCCGCACGTAGACCTTTAGC
hTGF-b1 Fwd	GACACGCAGTACAGCAAGGT
hTGF-b1 Rv	TGACACAGAGATCCGCAGTC
hTNFA Fwd	GCCTCTTCTCCTTCCTGATCG
hTNFA Rv	CAGCTTGAGGGTTTGCTACA
hTYRO3 Fwd	CGGTAGAAGGTGTGCCATTTT
hTYRO3 Rv	CGATCTTCGTAGTTCCTCTCCAC
hVEGFA Fwd	CGCAGCTACTGCCATCCAAT
hVEGFA Rv	GTGAGGTTTGATCCGCATAATCT

Supplementary S2 Table: anti-human antibodies.

Conjugate	Against	Cat no.	Clone	Host Species	Company
Alexa Fluor 488	CD206	321114/321113	15-2	Mouse IgG1, κ	Biolegend
Alexa Fluor 488	Akt (pS473)	560404	M89-61	Mouse IgG1, κ	BD Biosciences
Alexa Fluor 488	ERK1/2 (pT202/pY204)	612592	20A	Mouse IgG1	BD Biosciences
Alexa Fluor 647	CD64	305012	10,1	Mouse IgG1, κ	Biolegend
APC/Cy7	CD11b	101226	M1/70	Rat IgG2b, κ	Biolegend
FITC	Bcl-2	BMS1028FI	Bcl-2/100	Mouse IgG1, κ	ebioscience
FITC	HLA-DR	307632	L243	Mouse IgG2a, κ	Biolegend
PE	CD80	305207/8	2D10	Mouse IgG1, κ	Biolegend
PE/Cy7	CD14	325618	HCD14	Mouse IgG1, κ	Biolegend
PE/Cy7	CD44	103029	IM7	Rat IgG2b, κ	Biolegend
PerCP/Cy5.5	CD163	333607/333608	GHI/61	Mouse IgG1, κ	Biolegend

1.2 Supplementary Figures

Supplementary Figure S1

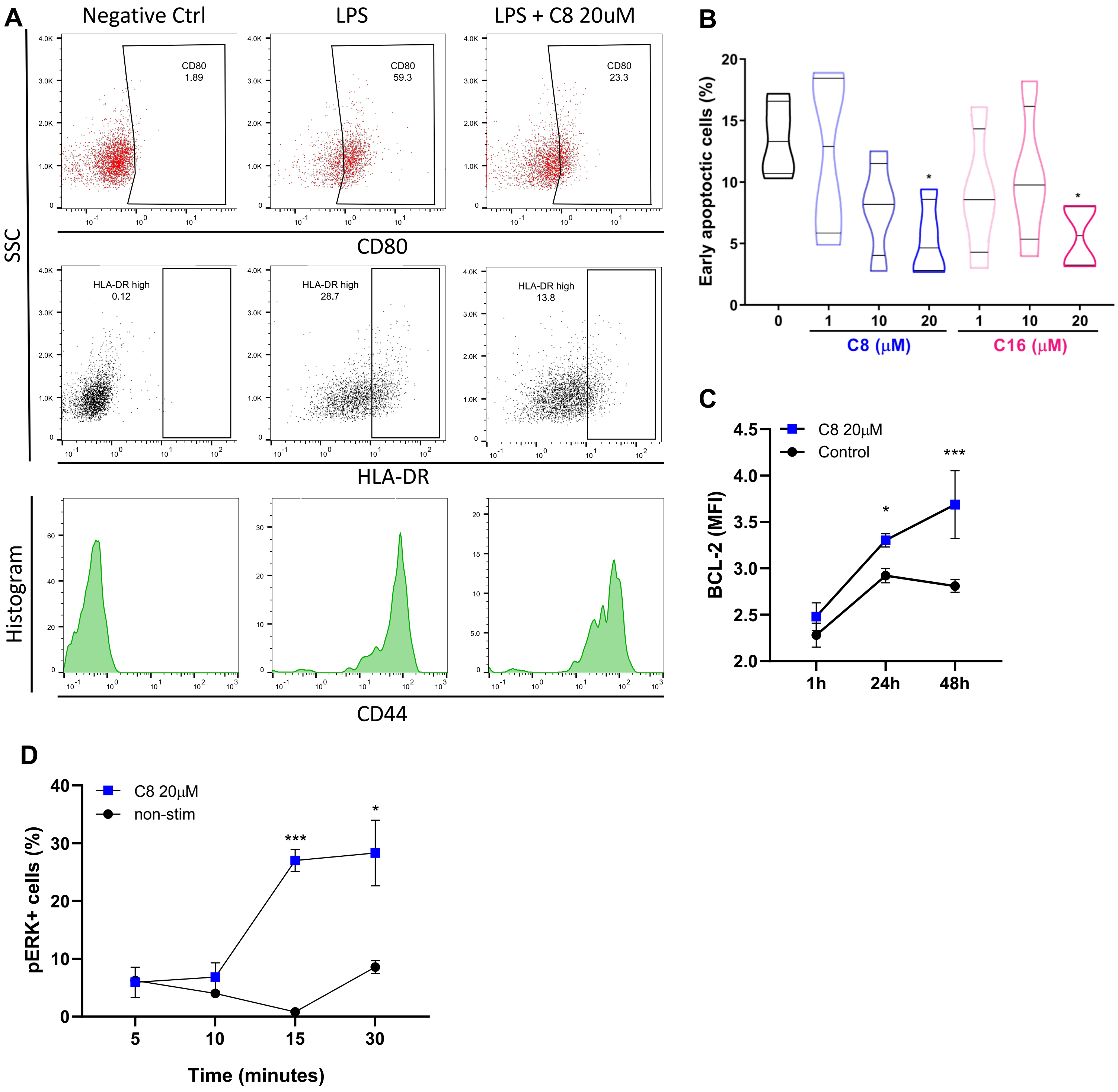

Supplementary Figure S1. C1P prevented apoptosis, induced BCL2 expression and triggered ERK phosphorylation in human monocytes. A) Representative flow cytometry dotplot comparing expression levels of CD80, HLA-DR and CD44 on monocytes stimulated with 10 ng/mL of LPS and 10 ng/mL of LPS plus 20μM of C8-C1P ceramide. **B)** Early apoptotic monocytes (Annexin V$^+$ Zombie Violet$^-$) after 24h of culture with 10 ng/mL of LPS and different concentrations of C8-C1P and C16-C1P. **C)** BCL-2 expression on monocytes treated with C8-C1P 20μM after 24 and 48 h compared to unstimulated control. **D)** Percentage of phosphoERK$^+$ monocytes after 3 h of starvation and stimulated with C8-C1P 20μM during 5 to 30 minutes compared with non-stimulated control. One-way pared ANOVA and Dunnett's post hoc test for B, two-way paired ANOVA and Fisher's post hoc test for C-D statistical significances *$p<0.05$, ***$p<0.005$.

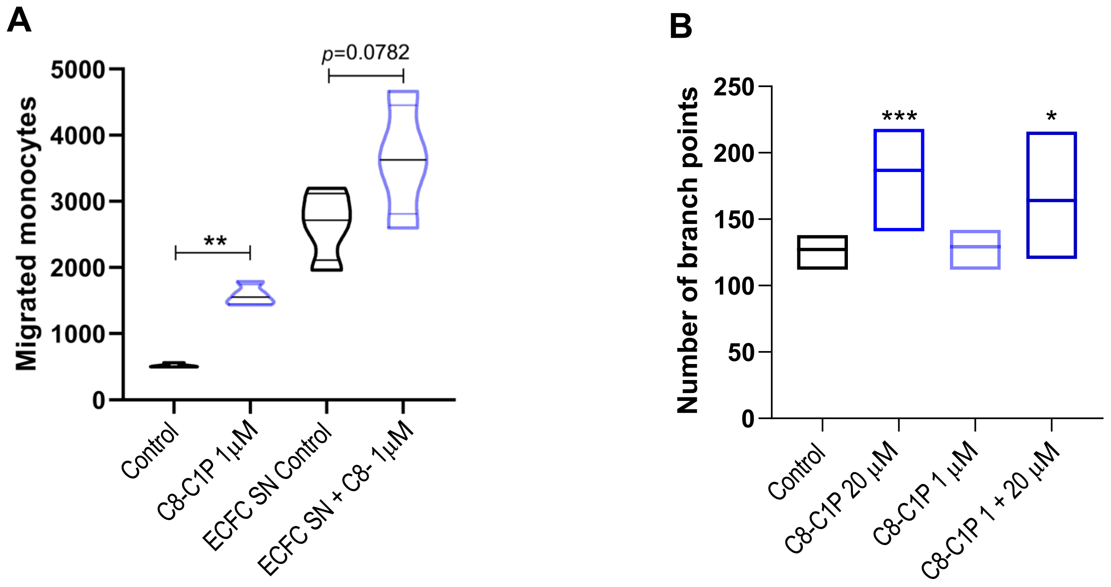

Supplementary figure S2. Monocytes migrate in response to low doses of C8-C1P while high doses conferred pro-angiogenic properties to monocytes-derived Macrophages (MDM). A) Number of monocytes that migrated through transwell membrane (8.0-μm pore polycarbonate membrane inserts) in response to control culture media (2% of FBS) or C8-C1P (1μM) stimulation, after 24h. Additionally, the chemotaxis assay was performed using the supernatant (SN) of hECFCs control or C8-C1P (1μM) stimulated cells. The number of migrated monocytes was determined by counting under a high-power microscope. **B)** Capillary-like tubule formation assay was performed culturing hECFC with 90% of reboosted MDM supernatants derived from C8-C1P-induced MDM at the following concentrations, 1, 20 or 1+20 μM as well as MDM control. The addition of two C1P concentration implies two administrations separated by 2h in between. One-way paired ANOVA and Fisher's post hoc test, statistical significances $*p<0.05$, $***p<0.005$. Independent data from each experiment, n = 4–7.

C

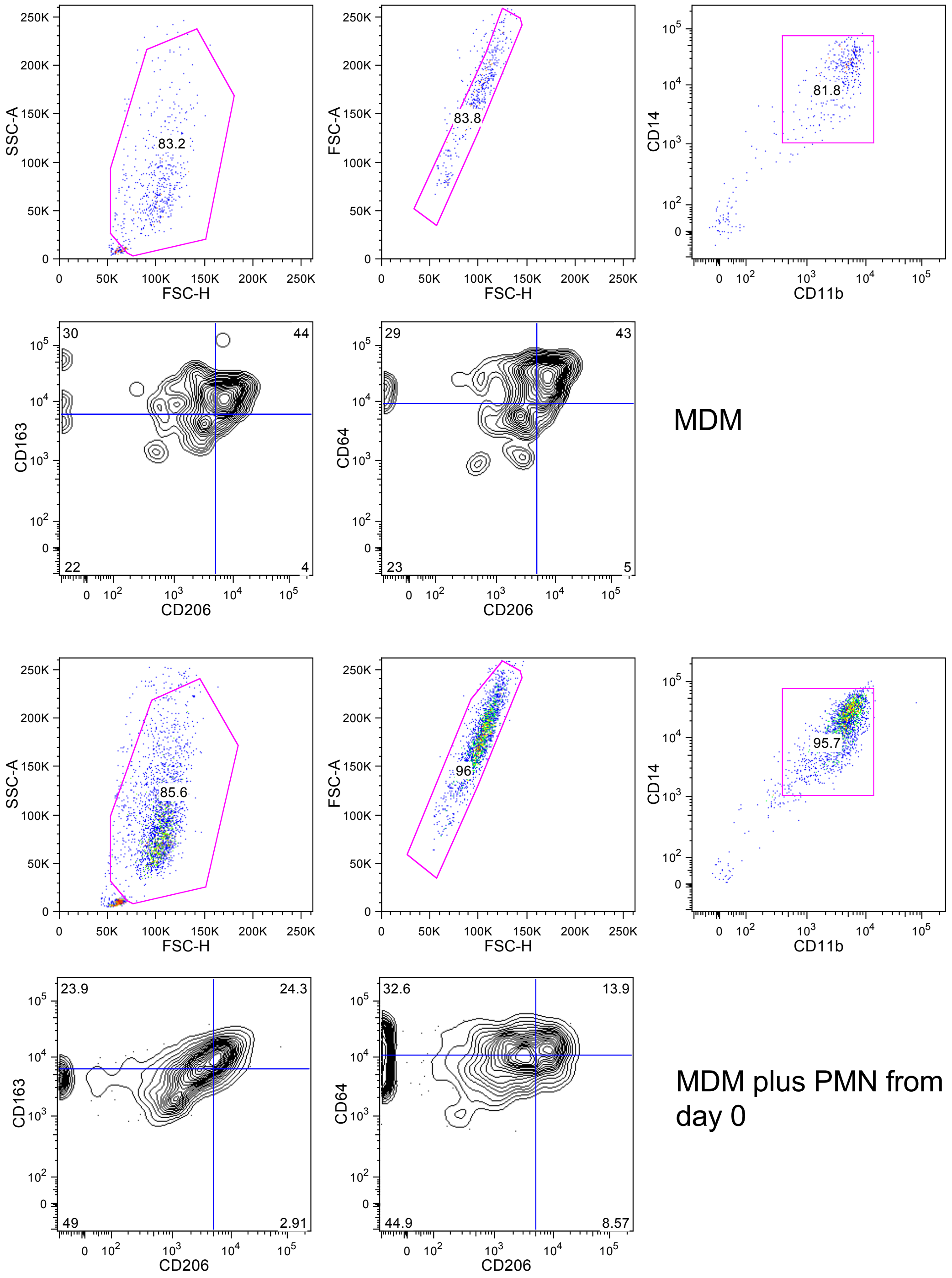

Supplementary figure S2 continue. C) Gating strategy of control MDM after 7 days of differentiation and MDM differentiated in presence of pro-inflammatory stimulus employing necrotic neutrophils (PMN) from day 0. The culture were harvest at day 7. Representative dot plots showing cells gating in $CD14^{+}CD11b^{+}$ cells and then the expression of CD206 vs CD163 and CD64 vs CD206 markers are shown in each culture condition. MDM = monocyte-derived macrophages.

A

Control: IL4, FOXO1, GAS6, CD36, IRF1, TYRO3, TGFB1, PDGFA, IGF1

C8 1 uM: VEGFA, MER (MERTK), PPARG, LXRA (NR1H3), IL10, CD163 and MRC1

C8 20 uM: VEGFA, MER (MERTK), PPARG, LXRA (NR1H3), IL10, PDGFA, FGF2

C8 1 + 20 uM: VEGFA, MER (MERTK), PPARG, LXRA (NR1H3), IL10, PDGFA, FGF2, CD163 and MRC1

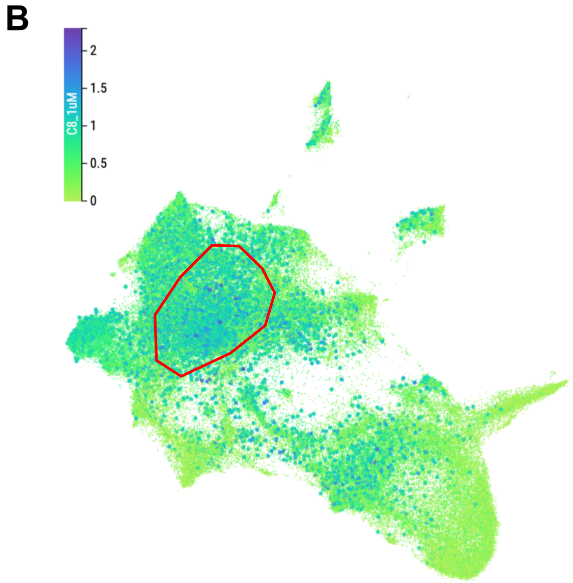

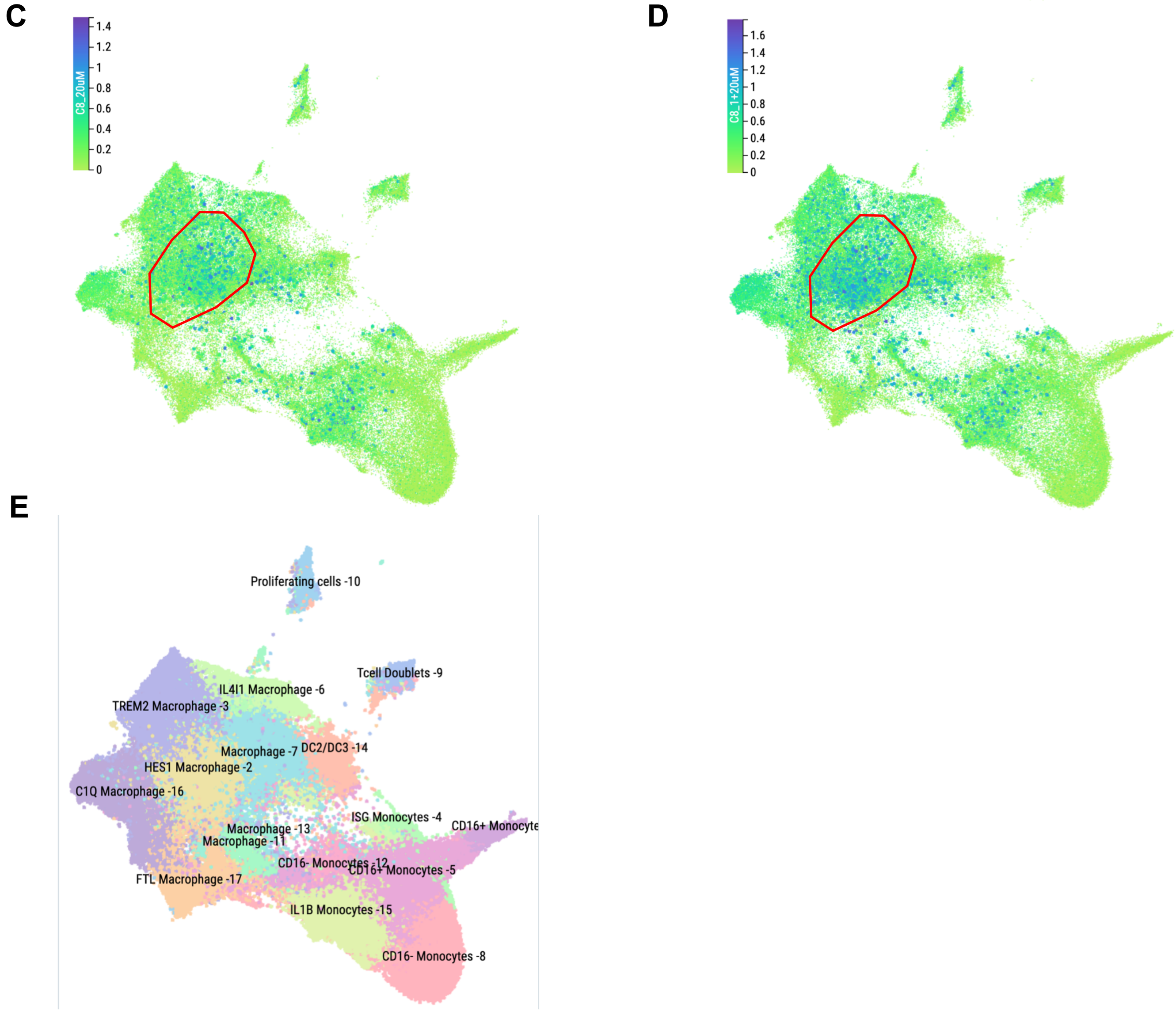

Supplementary Figure S3. Representative maps for C-8-C1P-induced MDM visualized within the MoMacverse framework. **A)** The preponderant gene expression profile of each condition are indicated. **B-D)** The distribution map for C8-induced MDM showing those cells that most strongly express their respective gene signatures for C8_1uM, C8_20uM, C8_1+20uM respectively. **E)** The specific localization of a given lineage into the MoMacverse map. Each cluster is defined with a name and number as it was described by Mulder et al. (19) and https://macroverse.gustaveroussy.fr/2021_MoMac_VERSE/.

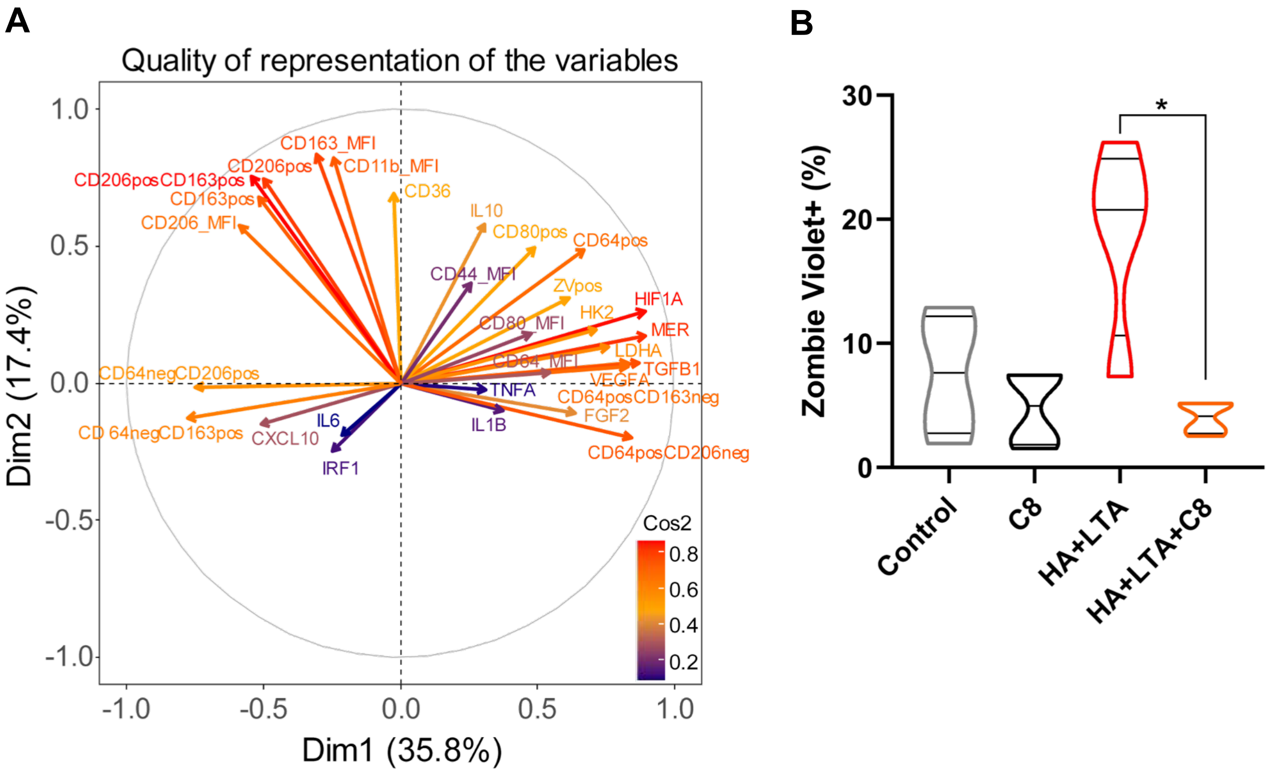

Supplementary Figure S4. C8-C1P restrains pro-inflammatory M1 program induced by LTA and HA and reduces mortality culture in MDM. A) Circle of correlation with vectorized variables (expression parameters) showing their interrelationship and colored by Cos2 values. **B)** C8-C1P-treatment reduced cell death in Macrophages induced by the pro-inflammatory stimuli HA + LTA measured by the percentage of Zombie violet $^{+}$ cells.

C8 = C8-Ceramide 1- Phosphate (20µM), LTA = Lipoteichoic acid (20ug/ml), HA = low molecular weight hyaluronic acid (100ug/ml). One-way paired ANOVA and Fisher's post hoc test, statistical significances *p<0.05.
